# Coexisting Cirrhosis Worsens Inpatient Outcomes in Patients With Infective Endocarditis: A Cross-Sectional Analysis of the National Inpatient Sample 2013-2014

**DOI:** 10.7759/cureus.11826

**Published:** 2020-12-01

**Authors:** Mohammad Jamil, Asim Kichloo, Ronak G Soni, Shakeel Jamal, Muhammad Zatmar Khan, Mitra Patel, Michael S Albosta, Michael Aljadah, Beth Bailey, Jagmeet Singh, Khalil Kanjwal

**Affiliations:** 1 Internal Medicine, Central Michigan University College of Medicine, Saginaw, USA; 2 Internal Medicine, University of Toledo College of Medicine, Toledo, USA; 3 Internal Medicine, Medical College of Wisconsin, Milwaukee, USA; 4 Nephrology, Geisinger Commonwealth School of Medicine, Scranton, USA; 5 Cardiology, McLaren Greater Lansing, Lansing, USA

**Keywords:** cirrhosis, infective endocarditis, liver, cardiology, perioperative mortality

## Abstract

Introduction

Cirrhosis is known to be an important prognostic factor in determining morbidity and mortality in preoperative cardiac risk assessment for cardiac surgery. Data is limited on outcomes in patients with infective endocarditis (IE) and comorbid liver cirrhosis. The objective of our study is to evaluate the clinical outcomes in patients suffering from IE both with and without underlying liver cirrhosis as well as to determine rates of in-hospital mortality and factors that contribute to this outcome.

Hypothesis

Liver cirrhosis worsens clinical outcomes in patients with IE.

Materials and methods

Patients with a principal diagnosis of IE with and without liver cirrhosis were identified by querying the Healthcare Cost and Utilization (HCUP) database, specifically the National Inpatient Sample for the years 2013 and 2014 using International Classification of Diseases, Ninth Revision (ICD-9) codes.

Results

During 2013 and 2014, a total of 17,952 patients were admitted with a diagnosis of IE, out of whom 780 had concurrent liver cirrhosis. There was increased in-hospital mortality [15.6% vs 10.2%, aOR = 1.57 (1.27-1.93)], acute kidney injury [41.4% vs 32.6%, aOR = 1.45 (1.24-1.69)], and hematologic complications [32.1 vs 14.7%, aOR = 2.87 (2.44-3.37)] in patients with IE with liver cirrhosis when compared to patients with IE without liver cirrhosis. Patients having IE without liver cirrhosis underwent an increased number of interventions, i.e. aortic (7.2 vs 3.7%, aOR = 0.51 (0.34-0.76)) and mitral (4.9% vs 3.4%, aOR = 0.39 (0.23-0.69)) valvular replacements as compared to those with liver cirrhosis, which may explain the increased mortality seen in patients with liver cirrhosis.

Conclusion

Liver cirrhosis is an important prognostic risk factor for in-hospital mortality in patients with IE. The coagulopathic state in addition to increased rates of bleeding complications and renal dysfunction make these patients poor surgical candidates thus contributing to higher mortality. Further research into the individual risk factors contributing to the increased mortality rates in patients with IE and cirrhosis is required.

## Introduction

Patients with cirrhosis are predisposed to develop a range of bacterial infections, primarily due to compromised immune function [1]. This results from decreased phagocytic ability, decreased opsonization, leukocyte dysregulation, as well as several iatrogenic factors, such as predisposition to undergoing invasive therapeutic, and diagnostic procedures such as gastrointestinal endoscopies, paracentesis, and trans-jugular intrahepatic portosystemic shunt (TIPS) [2-3]. Some of the common bacterial infections in cirrhotic patients that have been associated with increased morbidity and mortality are spontaneous bacterial peritonitis, pneumonia, and urinary tract infections [4].

Infective endocarditis (IE) is a rare but dreaded comorbidity in patients with liver cirrhosis and is associated with high morbidity and mortality [5]. Research studies and anecdotal case reports have suggested that several invasive therapeutic and diagnostic procedures, such as TIPS, band sclerotherapy for esophageal varices, upper gastrointestinal endoscopy, liver biopsy, and Sengstaken-Blakemore tube insertion in cirrhotic patients, increases the risk for IE [6-7]. These invasive procedures act as a portal for bacterial entry into the body. Staphylococcus aureus is the most common organism reported, followed by gram-negative bacilli of the intestinal tract [3]. In general, cirrhotic patients have increased mortality rates secondary to various factors such as coagulopathy resulting from the abnormal synthetic function of the liver and increased incidence of major bleeding. This coagulopathic state may predispose patients to an increased need for surgical interventions, such as heart valve replacement, and the elevated bleeding risk leads to higher overall morbidity in the IE patients when valvular surgeries are warranted [8]. Additionally, many cirrhotic patients have concurrent kidney dysfunction secondary to hepatorenal disease. This further adds to the poor prognosis of cirrhotic patients [9-10]. The Child-Pugh and model for end-stage liver disease (MELD) scores are widely used scoring systems for cirrhotic patients to assess prognosis. Studies have suggested that patients with increased Child-Pugh and MELD scores are poor surgical candidates due to increased intraoperative mortality risk [8,11-12].

Limited data are available regarding the outcomes of IE in patients with liver cirrhosis. Therefore, we aimed to evaluate in-hospital outcomes in IE patients both with and without liver cirrhosis.

## Materials and methods

Data source

The National Inpatient Sample (NIS), considered one of the largest publicly available databases in the USA, is part of the Healthcare Cost and Utilization Project (HCUP) and is maintained by the Agency for Health Care Quality and Research (AHQR). It is considered one of the most useful databases to study outcomes and trends of various procedures and diseases. It consists of de-identified data collected from 20% of community hospitals in 46 U.S. states. Each hospitalization includes one primary diagnosis and up to 29 secondary diagnoses and 15 procedures using the International Clinical Modification Codes 9 and 10 (ICD-9 and ICD-10). The database includes variables for admitting diagnoses, discharge diagnoses, length of hospital stay and incurred costs, demographic characteristics (i.e. age, race, gender), as well as the type and location of the healthcare facility.

Methods and outcomes

We evaluated NIS patients over the age of 18 years with the diagnosis of infective endocarditis during the years 2013 and 2014 using ICD-9 diagnostic codes. The ICD-9 codes used for the principal diagnoses of IE were as follows: 421.0, 421.9, 036.42, 098.84, 112.81, and 115.4 [13]. Endocarditis due to syphilis, rheumatic heart disease-related IE, lupus, and other non-infectious causes was excluded. Patients with liver cirrhosis were identified using the ICD-9 codes as follows: 571.2, 571.5, 571.6, 572.3, 572.2, 572.4, 456.0, 456.20, 578.0, 578.1, 578.9, 572.4 [13]. Hospitalizations were divided into two groups: IE with liver cirrhosis and IE without liver cirrhosis. Hospitalizations with missing demographics, admission or discharge diagnoses, length of stay, or mortality were excluded. The final set of variables used for analysis included age, race, gender, type of healthcare facility, income quartile, type of insurance, and comorbidities, including chronic kidney disease (CKD), diabetes mellitus (DM), hypertension, peripheral arterial disease (PAD), and anemia. The study included patients of Caucasian, African-American, and Hispanic ethnicity.

The primary study outcome was in-hospital mortality. The secondary outcomes examined were cardiogenic shock, metabolic acidosis, sepsis, hepatic complications, aortic valve replacement (AVR), mitral valve replacement (MVR), tricuspid valve surgery, pulmonary valve surgery, bleeding complications, stroke, acute kidney injury (AKI), length of hospital stay, and cost of hospital stay.

Statistical analyses 

The Statistical Package for the Social Sciences (SPSS) software (IBM Corp. Armonk, NY) was used to perform statistical analyses. Chi-square tests and independent t-tests were used to identify differences between hospitalizations for IE with comorbid liver cirrhosis and hospitalizations for IE without liver cirrhosis. Logistic regression models were used to calculate odds ratios for the outcomes between the two study groups, both adjusted and unadjusted for confounding variables. A p-value of <0.05 was considered statistically significant. Analyses were audited using the checklist provided by HCUP to assess and ensure data analyses and interpretations conformed to standards.

## Results

A total of 17,952 hospitalizations in the NIS sample from 2013-2014 included a diagnosis of IE, of which liver cirrhosis was identified in 780 (n=780). The study population was divided into two groups: patients with IE and co-morbid liver cirrhosis (n=780), and patients with IE without liver cirrhosis (n=17,172). Table 1 shows background characteristics by study group. It can be well appreciated that there was no significant age difference between the two groups (57.4±19.7 vs 56.9±12.2 years) with a p-value of 0.297. There was a greater percentage of females in the group having IE without liver cirrhosis (39.6% vs 32.7%). There were significantly more whites in the group having IE without liver cirrhosis as compared to IE with liver cirrhosis (72.2% vs 64.7%). For Black and Hispanic patients, the numbers were as follows: 15.0% vs. 11.9% and 8.0% vs. 16.2%, respectively. There was a greater percentage of diabetics in the IE with liver cirrhosis group (20.6% vs 15.4%). A similar trend was noted for chronic kidney disease (7.1% vs 5.7%).

**Table 1:** Characteristics of patients with infective endocarditis with coexisting liver cirrhosis

Characteristics	Infective endocarditis without liver cirrhosis	Infective endocarditis with liver cirrhosis	p-value
Number of patients (n)	n=17, 172	n=780	
Age- mean (SD), y	57.4 ± 19.7	56.9 ± 12.2	0.297
Female	39.6%	32.7%	<0.001
Race			
White	72.2%	64.7%	<0.001
Black	15.0%	11.9%	
Hispanic	8.0%	16.2%	
Hypertension	28.2%	21.9%	<0.001
Diabetes mellitus	15.4%	20.6%	<0.001
Chronic kidney disease	5.7%	7.1%	0.103
Atrial fibrillation	23.8%	17.4%	<0.001
Anemia	18.8%	16.4%	0.098
Peripheral arterial disease	16.1%	11.8%	0.001
Teaching hospital	30.0%	29.8%	0.842
Rural location	37.1%	37.3%	0.735
Large hospital bed size	25.6%	25.2%	0.564
Primary payer			
Medicare / Medicaid	68.8%	71.2%	0.241
Private Insurance	21.0%	18.5%	

**Figure 1:**
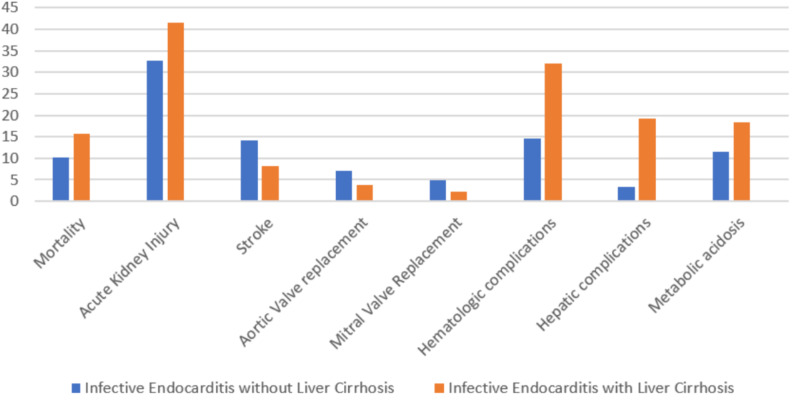
Outcomes of infective endocarditis with and without liver cirrhosis

Both Figure 1 and Table 2 demonstrate the comparison of the two study groups for outcomes of interest. It can be well-appreciated that patients with IE and liver cirrhosis had significantly higher rates of in-hospital mortality [a-OR = 1.57 (1.27-1.93)]. No significant difference was found for the length of hospital stay [a-OR = 0.95 (0.81-1.11)]. Patients with IE and liver cirrhosis had a nearly three-fold increased risk of hematologic complications [a-OR = 2.87 (2.44-3.37)]. Similar results were noted for acute kidney injury [a-OR = 1.45 (1.24-1.69)], hepatic complications [a-OR = 6.96 (5.65-8.56)], and metabolic acidosis [a-OR = 1.71 (1.41-2.08)]. Our study showed that the incidence of stroke was significantly decreased in the IE with liver cirrhosis group [a-OR = 0.58 (0.44-0.75)]. Patients with IE with liver cirrhosis had comparatively fewer valve replacement procedures, with AVR occurring at [a-OR = 0.51 (0.34-0.76)] and MVR occurring at [a-OR = 0.39 (0.23-0.69)]. Tricuspid valve (TV) surgery [a-OR = 0.42 (0.17-1.02)] and pulmonary valve (PV) surgery [a-OR = 0.71 (0.10-5.25)] were also performed less frequently in the cirrhosis group, however, the result was not statistically significant.

**Table 2:** Inpatient outcomes in patients with infective endocarditis with and without liver cirrhosis Note: Adjusted for gender, race, income, payor, hypertension, diabetes mellitus, AFIB, anemia, and peripheral arterial disease. a-Predicting length of stay greater than two weeks b- Predicting cost greater than $150,000 AFIB: atrial fibrillation

In-Hospital Outcomes	Infective endocarditis without liver cirrhosis	Infective endocarditis with liver cirrhosis	a-OR
In-Hospital Death	10.2%	15.6%	1.57 (1.27-1.93)
Stroke	14.1%	8.3%	0.58 (0.44-0.75)
Acute Kidney Injury	32.6%	41.4%	1.45 (1.24-1.69)
Aortic valve replacement	7.2%	3.7%	0.51 (0.34-0.76)
Mitral Valve replacement	4.9%	2.3%	0.39 (0.23-0.69)
New Dialysis	14.8%	16.5%	0.95 (0.76-1.18)
Cardiogenic shock	3.5%	3.6%	0.97 (0.64-1.46)
Cardiac arrest	2.5%	2.1%	0.80 (0.47-1.35)
Hematologic complications	14.7%	32.1%	2.87 (2.44-3.37)
Hepatic complications	3.3%	19.2%	6.96 (5.65-8.56)
Metabolic acidosis	11.6%	18.3%	1.71 (1.41-2.08)
Sepsis	44.4%	44.9%	0.97 (0.84-1.13)
Length of stay (days)	14.0 ± 15.6	13.5 ± 13.9	0.95 (0.81 – 1.11)^a^
Mean Cost ($)	$153,346 ± $230,220	$138,153 ± $175,953	0.88 (0.74-1.04)^b^
Tricuspid Valve Surgery	1.7%	0.8%	0.42 (0.17-1.02)
Pulmonary Valve Surgery	0.2%	0.1%	0.71 (0.10-5.25)

## Discussion

Cirrhosis is a common global ailment and is prevalent worldwide. According to a study by Behera, the global prevalence of cirrhosis is around 4.5%-9.5% [14]. Non-alcoholic steatohepatitis (NASH) and alcoholism are a few of the leading causes of cirrhosis in developed countries, whereas viral hepatitis is a leading cause of cirrhosis in underdeveloped countries [15-16]. One of the rare and infrequently reported infections associated with cirrhosis is IE. Whether liver cirrhosis acts as a risk factor for the development of infective endocarditis has not been thoroughly studied. According to one study, cirrhosis has been observed in 10% of cases of bacterial IE [17]. Of these cases, 45% are nosocomial, which is much higher than bacterial IE in patients without liver cirrhosis [17].

Our study particularly focused on inpatient outcomes for IE patients with and without cirrhosis. Increased rates of in-hospital mortality was one of the striking results observed in our study. IE patients with cirrhosis were noted to have a roughly 1.5 fold increase in hospital mortality as compared to patients without cirrhosis. As discussed above, this could be explained by the immunocompromised state of cirrhotic patients along with the multiple comorbidities often seen in these patients, including chronic kidney disease, cardiovascular disease, and coagulopathy. In addition, these patients are poor surgical candidates at baseline [8,11-12,18]. Therefore, they may not be able to undergo lifesaving procedures such as heart valve replacement. This is corroborated by the findings in our analysis that suggest that the rates of valvular surgeries were significantly lower in cirrhotic patients. Similarly, hematological complications were almost three times higher in IE patients with cirrhosis compared to the patients without cirrhosis. Cirrhosis patients have a significantly reduced synthetic function of the liver, which affects the formation of several clotting factors [8,19]. As a result, a significant number of cirrhotic patients are coagulopathic on presentation. This leads to a significantly increased risk of undergoing surgical procedures, including hematological and vascular complications as shown in this study.

Patients with cirrhosis are at high risk for encephalopathy, especially when their disease is complicated by sepsis. We also noticed that rates of hepatic complications were significantly higher in IE patients with cirrhosis. One surprising finding observed in this study was lower rates of stroke in IE patients with cirrhosis. For unknown reasons, the prevalence of atrial fibrillation was higher in the IE patients without cirrhosis, which may explain the higher rate of stroke in the patients without cirrhosis. Stroke in IE may result from embolization of the valvular vegetations [18]. Because of this, the role of anticoagulation is questionable and even possibly counterproductive [20-21]. There are no major randomized controlled studies available that evaluate anticoagulation in IE. Therefore, the use of anticoagulative therapies in cirrhotic patients with IE to decrease the incidence of stroke is highly questionable.

Regarding quality index data, such as the length and cost of hospitalization, no statistically significant difference was noted between the two groups. IE patients with cirrhosis have multiple comorbidities at baseline. High mortality and palliative management may be a potential contributing factor providing an explanation for the unexpected finding of a similar length of stay found in the analysis. An overall trend of a lower cost of stay for the cirrhosis patients was observed, however, this finding is not statistically significant. This could be possibly explained by conservative and/or palliative management in cirrhotic patients with multiple comorbidities with poor prognosis versus more aggressive management and the undertaking of surgical procedures if required in the patients without cirrhosis.

There are limitations to the utilization of the Healthcare Utilization Project database, including errors in relation to the ICD-9 and ICD-10 coding systems. In order to prevent this, we have utilized codes that have been validated in previous studies. We have performed a retrospective analysis and given insight into an association between two conditions rather than attempting to prove causation between these conditions and the studied outcomes. An additional limitation is that the ICD coding system is unable to identify when patients are readmitted with the same condition. Because of this, every admission is considered a separate case and, therefore, a new patient encounter.

## Conclusions

Liver cirrhosis is an important prognostic risk factor for in-hospital mortality in patients with concurrent IE. Patients with IE and cirrhosis have higher rates of coagulopathy, bleeding complications, hepatic complications, and renal dysfunction that may contribute to higher mortality. In addition, patients suffering from cirrhosis and IE are significantly less likely to undergo valvular replacement surgery, likely due to an increased risk of undergoing surgical procedures in these patients. Interestingly, patients with IE and cirrhosis were less likely to suffer from a stroke than patients having IE without cirrhosis. Finally, there was no significant difference in both the length and cost of hospitalization between groups. Further research is required to evaluate factors that lead to worse clinical outcomes in patients suffering infective endocarditis with concurrent liver cirrhosis.

## Appendices

**Table 3:** ICD codes used for primary and secondary outcomes

Variable	Code
Infective Endocarditis	Acute and subacute bacterial endocarditis – 4210; Acute and subacute IE in disease classified elsewhere e.g. Q fever – 4211; Acute endocarditis, unspecified – 4219; Meningococcal endocarditis – 03642; Gonococcal endocarditis – 09884; Candidal endocarditis – 11281; Histoplasmosis endocarditis - 1154. * We excluded endocarditis due to syphilis, rheumatic heart disease (without infection), lupus, or other non-infectious causes.
Liver Cirrhosis	Diagnosis of liver cirrhosis – 571.2, 571.5, 571.6; Liver cirrhosis with or without: Portal hypertension - 572.3; Ascites – 789.59; Hepatic encephalopathy – 572.2; Upper gastrointestinal (GI) bleed - 456.0, 456.20, 578.0, 578.1, 578.9; Hepatorenal syndrome – 572.4
Tricuspid Valve Surgery	35.14, 35.27, 35.28
Pulmonary Valve Surgery	35.13, 35.25, 35.26
Heart block	First-degree heart block – 426.11; Mobitz type II second degree heart block – 426.12; Other second-degree heart block – 426.13; Third-degree heart block - 426.0
Aortic valve replacement	Replacement of aortic valve with tissue graft – 35.21; Other replacement of aortic valve – 35.22
Mitral valve replacement	Replacement of mitral valve with tissue graft – 35.23; Other replacement of mitral valve – 35.24
Cardiogenic Shock	785.51
Cardiac Arrest	427.5
Stroke	Cerebral artery occlusion, unspecified with cerebral infarction – 434.91; Subarachnoid hemorrhage – 430; Intracerebral hemorrhage – 431; Nontraumatic extradural hemorrhage – 432.0; Subdural hemorrhage – 432.1; Unspecified intracranial hemorrhage – 432.9; Cerebral thrombus with infarction – 433.01; Occlusion of carotid artery, with cerebral infarction – 433.11; Occlusion of vertebral artery with cerebral infarction – 433.21; Occlusion of bilateral precerebral arteries with infarction – 433.31; Occlusion of other precerebral arteries with cerebral infarction – 433.81; Occlusion of unspecified precerebral arteries with infarction – 433.91; Cerebral infarction without mention of cerebral infarction – 434.00; Cerebral infarction with cerebral infarction – 434.01; Cerebral embolism without mention of cerebral infarction – 434.10; Cerebral embolism with cerebral infarction – 434.11; Acute cerebral vascular disease, ill-defined – 436; Iatrogenic cerebral infarction – 997.02
Acute kidney injury	Acute kidney injury with tubular necrosis – 584.5; Acute kidney injury with lesion of renal cortical necrosis – 584.6; Acute kidney injury, other specified pathological lesion in kidney – 584.8; Acute kidney injury, unspecified – 584.9
Acute kidney injury requiring dialysis	Hemodialysis (in addition to acute kidney injury) – 39.95; End-stage renal disease – excluded – 585.6
Pacemaker implantation	Implantation of CRT-P, total system – 00.50; Implantation of CRT-D, total system – 00.51; Implantation of coronary sinus lead – 00.52; Implantation of CRT-P generator only – 00.53; Implantation of CRT-D generator only – 00.54; Initial insertion of lead, NOS – 37.70; Initial insertion of lead, ventricle – 37.71; Initial insertion of lead, ventricle, and atrium – 37.72; Initial insertion of lead, atrium – 37.73; Initial insertion of permanent pacemaker, NOS – 37.80; Initial insertion of single-chamber pacemaker – 37.81; Initial insertion of single-chamber pacemaker, rate-responsive – 37.82; Initial insertion of dual-chamber pacemaker – 37.83; Implantation of ICD, total system – 37.94; Implantation of ICD, generator only – 37.96
Sepsis	Sepsis – 995.91; Septicemia – 038; Severe sepsis – 995.92; Infection and inflammatory reaction due to indwelling urinary catheter – 996.64; Other and unspecified infection due to central venous catheter – 999.31; Bloodstream infection due to central venous catheter – 999.32
Hepatic	Hepatic necrosis – 570; Hepatic encephalopathy – 572.2; Unspecified hepatitis – 573.3
Hematologic	Thrombocytopenia – 287.3, 287.4, 287.5; Coagulopathy – 286.9
Metabolic/lactic acidosis	276.2
